# *Clostridium butyricum*-derived extracellular vesicles alleviate ETEC K88-induced intestinal injury by regulating Wnt/β-catenin signaling and gut microbiota

**DOI:** 10.1128/aem.00424-26

**Published:** 2026-08-28

**Authors:** Huiru Chen, Ningtao Chen, Shuang Liang, Lingyan Ma, Nana Jing, Yingping Xiao, Ying Ren, Haiqin Wu

**Affiliations:** 1 School of Animal Science and Nutritional Engineering, Wuhan Polytechnic University 554676, Wuhan, China; 2 State Key Laboratory for Quality and Safety of Agro-products, Zhejiang Academy of Agricultural Sciences 74561, Hangzhou, China; 3 Institute of Agro-product Safety and Nutrition, Zhejiang Academy of Agricultural Sciences 74561, Hangzhou, China; University of Illinois Urbana-Champaign, Urbana, Illinois, USA

**Keywords:** *Clostridium butyricum*, extracellular vesicles, Wnt/β-catenin signaling, gut microbiota, enterotoxigenic *Escherichia coli *K88

## Abstract

Probiotic-derived extracellular vesicles (EVs) serve as crucial mediators that alleviate intestinal barrier dysfunction. As a well-established probiotic, *Clostridium butyricum* (*Cb*) has been shown to ameliorate ETEC K88-induced intestinal injury. Nevertheless, the potential role of *Cb*-derived EVs (CbEVs) in ETEC K88-induced intestinal injury has not yet been elucidated. This study showed that CbEVs effectively ameliorated ETEC K88-induced ileal injury, as evidenced by improved ileal morphology and reduced levels of proinflammatory cytokines, diamine oxidase, and malondialdehyde. Mechanistically, CbEVs activated Wnt/β-catenin signaling, thereby accelerating the renewal of intestinal epithelial cell lineages, which is pivotal for maintaining intestinal homeostasis. In parallel, they also reshaped the ileal microbiota by restoring microbial balance. Collectively, these findings demonstrated that CbEVs alleviated ETEC K88-induced ileal barrier dysfunction by coordinating the restoration of intestinal stem cell (ISC) activity and gut microbiota composition, providing a theoretical basis for developing CbEV-based strategies to prevent ETEC-induced intestinal barrier dysfunction in both humans and animals.

**IMPORTANCE:**

ETEC infection-induced intestinal injury continues to pose a significant threat to human and livestock health, while effective preventive strategies remain limited. This study demonstrates that extracellular vesicles derived from the probiotic *Clostridium butyricum* accelerate intestinal epithelial regeneration by activating Wnt/β-catenin signaling and restoring gut microbiota homeostasis, thereby conferring protection against ETEC-induced intestinal injury. These findings provide a solid scientific basis for the development of *Clostridium butyricum*-derived extracellular vesicles as a promising non-viable probiotic (postbiotic) strategy to prevent intestinal infections, thereby offering a safer and more stable alternative to live bacteria in functional foods and feed additives.

## INTRODUCTION

*Escherichia coli *is one of the major enteric pathogens responsible for diarrhea in humans and livestock, which can lead to symptoms such as abdominal pain, diarrhea, fever, nausea, and vomiting. In severe cases, *E. coli* infection may result in dehydration, electrolyte imbalance, or even death (1–3). Such infections not only result in significant economic losses in the livestock industry but may also pose a risk to human health through contamination of the food chain. Among the various ETEC serotypes, ETEC K88 can disrupt intestinal barrier function by secreting enterotoxins, which lead to disturbances in electrolyte and water balance and diarrhea (4). Currently, antibiotic therapy remains the primary treatment for diarrhea caused by ETEC K88 infection (5). Nevertheless, the excessive use of antibiotics not only promotes antibiotic resistance in ETEC K88 but also disrupts the balance of the host intestinal microbiota (6). Particularly in the livestock industry, antibiotic residues in animal-derived foods can enter the human food chain and further pose a potential threat to human health. In addition, China has implemented a comprehensive “antibiotic ban” policy (7, 8). Therefore, there is an urgent need to develop an effective alternative to antibiotics for the prevention and treatment of *E. coli*-induced intestinal diseases.

*Clostridium butyricum* (*Cb*), a well-established probiotic (9), has been reported to combat intestinal pathogenic infections by restoring gut microbiota homeostasis (10), repairing intestinal barrier function (11, 12), and modulating the immune system (13). Moreover, *Cb* has also been extensively used to treat other non-infectious intestinal diseases (14). Extracellular vesicles (EVs) derived from bacteria are essential signaling agents in gut microbiota-host communication, primarily involved in modulating gut microecology, host immune responses, and intestinal barrier function (15, 16). Research has demonstrated that *Cb*-derived EVs (CbEVs) ameliorated acute colitis through various mechanisms (17, 18). Nevertheless, it remains unclear whether CbEVs can reverse the adverse effects of ETEC K88 infection on intestinal health.

Intestinal stem cells (ISCs), a group of cells with self-renewal capabilities and the ability to differentiate into multiple cell lineages, drive the continuous renewal of intestinal epithelial cells and contribute to the maintenance of intestinal homeostasis (19). The fate and function of ISCs are regulated by various signaling pathways, with Wnt/β-catenin signaling serving as a crucial regulator of ISC function, and its dysregulation is closely associated with intestinal diseases, including colorectal cancer and inflammatory bowel disease (20). Previous studies have demonstrated that *Cb* can alleviate ETEC K88-induced intestinal inflammatory injury and improve intestinal barrier function (14, 21). Nevertheless, whether CbEVs regulate ISC function via Wnt/β-catenin signaling to ameliorate ETEC K88-induced intestinal inflammatory injury remains unclear.

The intestine is the central organ for nutrient digestion and absorption and plays essential roles in detoxification and immune regulation. Therefore, its barrier integrity is essential for overall host health. As the terminal portion of the small intestine, the ileum is crucial for immune defense, nutrient uptake, and digestive regulation (22). It contains numerous transporters to facilitate the uptake of residual nutrients from the proximal small intestine, particularly vitamin B12 and bile acids (23). In addition, the submucosa and lamina propria of the ileum harbor numerous lymphoid structures, including Peyer’s patches and solitary lymphoid nodules, which are crucial for ileal immune function (24). Thus, the ileum is a primary target site of ETEC K88 infection, likely due to its unique histological and anatomical characteristics (25). Consequently, this study aimed to investigate the role and underlying mechanisms of CbEVs in ETEC K88-induced ileal inflammatory injury.

In this study, an ETEC K88-induced ileal inflammatory injury model was established in mice to evaluate the protective effects of CbEV pretreatment. We demonstrated that CbEVs alleviated ETEC K88-induced ileal inflammatory injury by activating Wnt/β-catenin signaling, promoting ISC proliferation and differentiation, and reshaping the gut microbiota. These findings demonstrate the preventive effects of CbEVs against ETEC K88-induced ileal barrier dysfunction and provide a theoretical basis to develop therapeutic strategies for preventing intestinal injury caused by *E. coli* infection.

## MATERIALS AND METHODS

### Isolation of CbEVs

Following the established protocols in our laboratory, CbEVs were obtained from the culture supernatant of *Cb* by ultracentrifugation (17). The isolated EVs were characterized by ultrastructural imaging and particle size distribution analysis, further confirming that they exhibited the characteristic morphology and size (17).

### Bacterial strains

The ETEC K88 strain was obtained from diarrheic piglet feces stored in our laboratory. Bacteria were cultured in Luria-Bertani (LB) broth (HB0128, Qingdao Hi-tech Industrial Park Hope Bio-technology Co., Ltd., Qingdao, China) under aerobic conditions (200 rpm, 37°C, 16 h). The bacterial cells were obtained by centrifugation (5,500 rpm, 10 min) and resuspended in sterile saline to a final concentration of 1 × 10^10^ CFU/mL for oral administration.

### Animals and experimental design

Twenty-four male C57BL/6 mice (7–8 weeks old) were obtained from Shanghai SLAC Laboratory Animal Co., Ltd., Shanghai, China. Throughout the experiment, the mice were housed under specific pathogen-free (SPF) conditions, with a humidity of 55% ± 3%, temperature of 23°C ± 1°C, and a 12-h light/dark cycle. During the experiment period, the mice had *ad libitum* access to standard rodent chow and sterile water, and bedding was changed twice weekly.

After a 7-day acclimatization period, the animals were randomly assigned to three experimental groups (n = 8 per group): (i) control group: received 200 µL of isotonic saline via oral gavage once daily for 25 days; (ii) model group (ETEC): received the same volume of isotonic saline daily for 21 days, followed by 200 µL of a bacterial suspension containing 2 × 10⁸ CFU of ETEC K88 once daily for 4 days; (iii) intervention group (EV + ETEC): received 50 µg of CbEVs suspended in 200 µL of isotonic saline daily for 21 days, followed by 200 µL of a bacterial suspension containing 2 × 10⁸ CFU of ETEC K88 once daily for 4 days. At the end of the experiment, the mice were euthanized by cervical dislocation. A portion of the ileal tissue was fixed in 4% paraformaldehyde (G1101, Wuhan Servicebio Technology Co., Ltd., Wuhan, China) for histological analysis, and the remaining ileal tissue, along with its contents, was snap-frozen and stored at −80°C for subsequent molecular biology experiments.

### Histological analysis

For histomorphological assessment, ileal tissue samples were first fixed in 4% paraformaldehyde (G1101, Wuhan Servicebio Technology Co., Ltd., Wuhan, China), followed by paraffin embedding. Serial 4-µm sections were prepared and stained with hematoxylin and eosin (H&E). Sections were examined under a light microscope, and villus height and crypt depth in the ileum were quantified using ImageJ software (National Institutes of Health, Bethesda, MD, USA).

### Quantification of inflammatory cytokines, intestinal barrier function, and oxidative stress indicators in the ileal tissue

Ileal tissue samples were homogenized on ice in RIPA buffer (P0013E, Beyotime Biotechnology Co., Ltd., Shanghai, China) supplemented with a protease inhibitor cocktail to prevent protein degradation during tissue disruption. Following centrifugation of the homogenates at 4°C and 12,000 × *g* for 15 min, the supernatants were carefully collected for subsequent analysis. The levels of TNF-α (EM0183, Wuhan Fine Biotech Co., Ltd., Wuhan, China), IL-6 (EM0121, Wuhan Fine Biotech Co., Ltd., Wuhan, China), IL-1β (EM0109, Wuhan Fine Biotech Co., Ltd., Wuhan, China), lipopolysaccharide (LPS) (EM2186, Wuhan Fine Biotech Co., Ltd., Wuhan, China), diamine oxidase (DAO) (EM0980, Wuhan Fine Biotech Co., Ltd., Wuhan, China), and malondialdehyde (MDA) (EU2577, Wuhan Fine Biotech Co., Ltd., Wuhan, China) in the ileal tissue were quantified using ELISA kits.

### Transcriptome analysis

Total RNA was isolated with TRIzol reagent (15596026CN, Thermo Fisher Scientific Co., Ltd., Shanghai, China) following the manufacturer’s protocol. RNA integrity and concentration were determined using Bioanalyzer 2100 and Agilent RNA 6000 Nano Kit (5067-1511, Agilent Technologies Co., Ltd., Beijing, China). Samples with an RNA integrity number (RIN) greater than 7.0 were used for subsequent library preparation. mRNA was enriched from total RNA using Dynabeads Oligo (dT)_25_ (61002, Thermo Fisher Scientific Co., Ltd., Shanghai, China) and then fragmented into short fragments. These RNA fragments were reverse-transcribed to generate cDNA, which served as the template for synthesis of U-labeled double-stranded DNA. The resulting DNA fragments underwent end repair and A-tailing prior to ligation with indexed adapters. Fragment selection was carried out using AMPureXP magnetic beads, followed by PCR amplification to generate the final sequencing libraries with an average insert size of 300 ± 50 bp. Sequencing was subsequently conducted on the Illumina NovaSeq 6000 platform using the 2 × 150 bp paired-end mode in accordance with the manufacturer’s instructions.

### Real-time quantitative PCR (RT-qPCR)

Total RNA was isolated from ileal tissue samples using the TransZol Up Kit (ET111-01-V2, Beijing TransGen Biotech Co., Ltd., Beijing, China). Subsequently, total RNA was reverse-transcribed into cDNA using the TransScript Uni All-in-One First-Strand cDNA Synthesis SuperMix Kit (One-Step gDNA Removal) (AU341-02, Beijing TransGen Biotech Co., Ltd., Beijing, China). Target genes were amplified on a real-time PCR system using PerfectStart Green qPCR SuperMix (AQ601-01-V2, Beijing TransGen Biotech Co., Ltd., Beijing, China) and gene-specific primers. Relative mRNA expression levels of the target genes were normalized to β-actin and calculated using the 2^−ΔΔCt^ method. The primer information is shown in Table S1.

### Immunofluorescence

Following deparaffinization and rehydration, paraffin sections were subjected to microwave-mediated antigen retrieval using citrate antigen retrieval buffer (pH = 6.0). After cooling to room temperature and washing three times with PBS, the sections were blocked with 3% BSA for 30 min. Subsequently, the sections were incubated overnight at 4°C with the following primary antibodies: Claudin-8 rabbit pAb (40-0700Z, 1:2,000, Thermo Fisher Scientific Co., Ltd., Shanghai, China), Non-phospho (Active) β-catenin rabbit mAb (#8814, 1:1,000, Cell Signaling Technology, Danvers, Massachusetts, USA), Olfm4 rabbit mAb (#39141, 1:400, Cell Signaling Technology, Danvers, Massachusetts, USA), Anti-lysozyme rabbit pAb (GB11345-50, 1:500, Wuhan Servicebio Technology Co., Ltd., Wuhan, China), Muc2 rabbit pAb (A14659, 1:200, ABclonal Technology Co., Ltd., Wuhan, China), and Keratin 20 rabbit mAb (#13063, 1:500, Cell Signaling Technology, Danvers, Massachusetts, USA). Following three washes with PBS, the sections were incubated with the corresponding fluorescent secondary antibodies for 50 min at room temperature in the dark. After washing the sections three times with PBS, the nuclei were counterstained with DAPI (G1012, Wuhan Servicebio Technology Co., Ltd., Wuhan, China) and incubated in the dark at room temperature for 10 min. After further PBS washing, the sections were mounted with anti-fade mounting medium and observed under a fluorescence microscope.

The relative fluorescence intensities of Claudin-8, β-catenin, and Krt20, as well as the numbers of Olfm4^＋^ and Lyz1^＋^ cells per crypt and Muc2^＋^ cells per villus, were analyzed using ImageJ software (National Institutes of Health, Bethesda, MD, USA). The relative fluorescence intensities of Claudin-8, β-catenin, and Krt20 were quantified and normalized to the DAPI fluorescence signal to minimize variations in epithelial cell numbers among images, whereas Olfm4^＋^ and Lyz1^＋^ cells were quantified as the number of positive cells per crypt, and Muc2^＋^ cells as the number of positive cells per villus.

### 16S rRNA gene sequencing

Total genomic DNA was isolated from ileal contents using the E.Z.N.A. Stool DNA Kit (D4015-00S, Omega Bio-tek, Norcross, GA, USA). DNA concentration and integrity were evaluated by spectrophotometry and agarose gel electrophoresis, respectively. The V3–V4 regions of the bacterial 16S rRNA gene were amplified by PCR using the forward primer 515F (5′-GTGCCAGCMGCCGCGGTAA-3′) and the reverse primer 907R (5′-CCGTCAATTCMTTTRAGTTT-3′). The amplified products were separated on 2% agarose gels, purified using the AxyPrep DNA Gel Extraction Kit (Axygen Biotechnology Co., Ltd., Hangzhou, China), and pooled at equal concentrations. Sequencing libraries were subsequently prepared with the NEBNext Ultra DNA Library Preparation Kit and sequenced on the Illumina MiSeq platform (Shanghai Lingen Biotechnology Co., Ltd., Shanghai, China). Raw sequencing data were further processed and analyzed using the QIIME 2 platform (version 2025.4).

### Statistical analysis

Quantitative data were presented as the mean ± SEM. Data visualization was performed using GraphPad Prism 9.5 (GraphPad Software, San Diego, CA, USA), and statistical analysis was conducted with SPSS 27.0 (International Business Machines Corporation, Armonk, NY, USA). For data sets with normal distribution and homogeneity of variance, one-way analysis of variance (ANOVA) was applied. Differences among groups were further evaluated using Fisher’s least significant difference (LSD) post hoc test. *P* < 0.05 was considered statistically significant.

For transcriptome data, DESeq2 was used for gene differential expression analysis, and differentially expressed genes (DEGs) were screened using the threshold criteria of |log2(fold change)| ≥1 and *P* < 0.05. Volcano plots, Kyoto Encyclopedia of Genes and Genomes (KEGG) enrichment analysis, heatmap, and gene set enrichment analysis (GSEA) were generated using the cloud-based tools provided by the OmicStudio Cloud Platform of LC-Bio Technology.

For 16S rRNA gene sequencing, α-diversity indices (Chao1, Shannon, and Simpson) were calculated using QIIME2. β-diversity was assessed by principal coordinates analysis (PCoA) based on Bray-Curtis distances, and differences among groups were analyzed by permutational multivariate analysis of variance (PERMANOVA). The differences in bacterial genus abundance and α-diversity indices between the two groups were analyzed using the Mann-Whitney U test. Linear discriminant analysis effect size (LEfSe) analysis was performed using the online LEfSe analysis tool provided by the Wekemo Bioincloud Platform to identify differential taxa with a linear discriminant analysis (LDA) score ≥ 2. Microbial functional predictions were conducted using PICRUSt2, followed by statistical analysis in STAMP.

Visualization of the integrative correlation analysis was performed using online tools provided by the OmicStudio Cloud Platform of LC-Bio Technology, including Circos plot, correlation network heatmap, and clustered correlation heatmap. Correlations were calculated using Spearman’s correlation coefficients, and *P* < 0.05 was considered statistically significant, with *P < 0.05, **P < 0.01, ***P < 0.001, and ns indicating *P* > 0.05.

## RESULTS

### CbEVs alleviate ileal injury induced by ETEC K88 in mice

**Fig 1:**
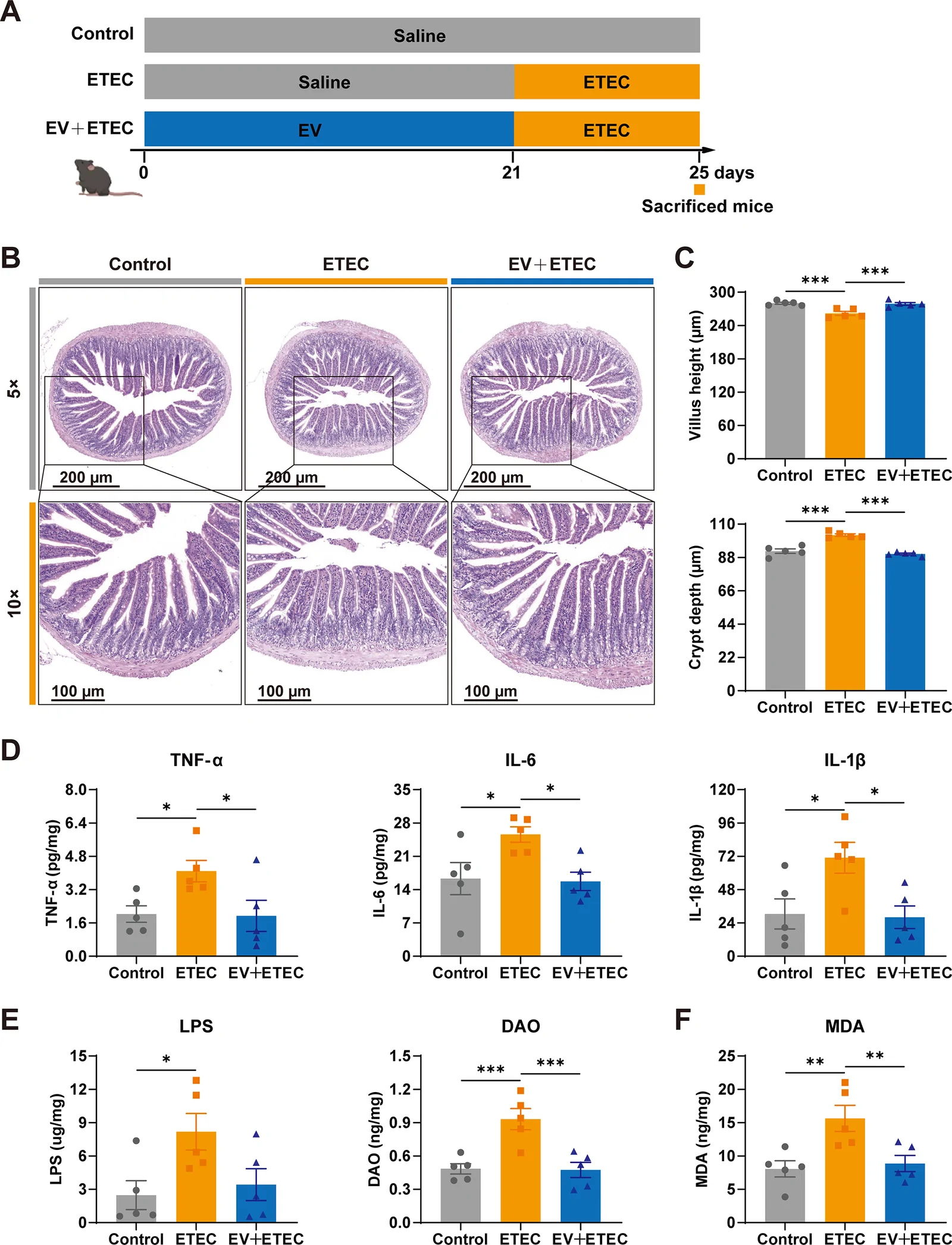
CbEVs alleviate ETEC K88-induced ileal injury in mice. (**A**) Schematic diagram of the experimental design. (**B**) Representative H&E staining images of ileal sections (scale bars = 100 and 200 µm). (**C**) Quantitative analysis of ileal villus height and crypt depth. (**D**) Levels of inflammatory cytokines TNF-α, IL-6, and IL-1β in ileal tissue were measured by ELISA. (**E**) Levels of intestinal barrier function-related indicators LPS and DAO in ileal tissue were measured by ELISA. (**F**) Levels of the oxidative stress indicator MDA in ileal tissue were measured by ELISA. All data are presented as the mean  ±  SEM, n = 5, *P ≤ 0.05, **P ≤ 0.01, ***P ≤ 0.001.

To investigate the effects of CbEVs on ETEC K88*-*induced ileal injury in mice, mice were pretreated with CbEVs before ETEC K88 infection (Fig. 1A). Histological analysis showed that ETEC K88 infection markedly reduced ileal villus height (*P* < 0.001) and increased crypt depth (*P* < 0.001), whereas CbEV pretreatment significantly attenuated these alterations (Fig. 1B and C). Furthermore, ELISA analysis revealed that ETEC K88 infection led to significant rises in the levels of TNF-α (*P* = 0.025), IL-6 (*P* = 0.020), IL-1β (*P* = 0.015), LPS (*P* = 0.047), DAO (*P* < 0.001), and MDA (*P* = 0.004) in ileal tissues. In contrast, CbEV treatment significantly reduced the levels of TNF-α (*P* = 0.021), IL-6 (*P* = 0.014), IL-1β (*P* = 0.011), DAO (*P* < 0.001), and MDA (*P* = 0.008), while no significant effect was observed on LPS levels (*P* = 0.117) (Fig. 1D through F). Collectively, these results suggest that CbEVs effectively alleviate ETEC K88*-*induced ileal injury and inflammation in mice.

### Effects of CbEVs on the ileal transcriptome profiles in ETEC K88 infection mice

**Fig 2:**
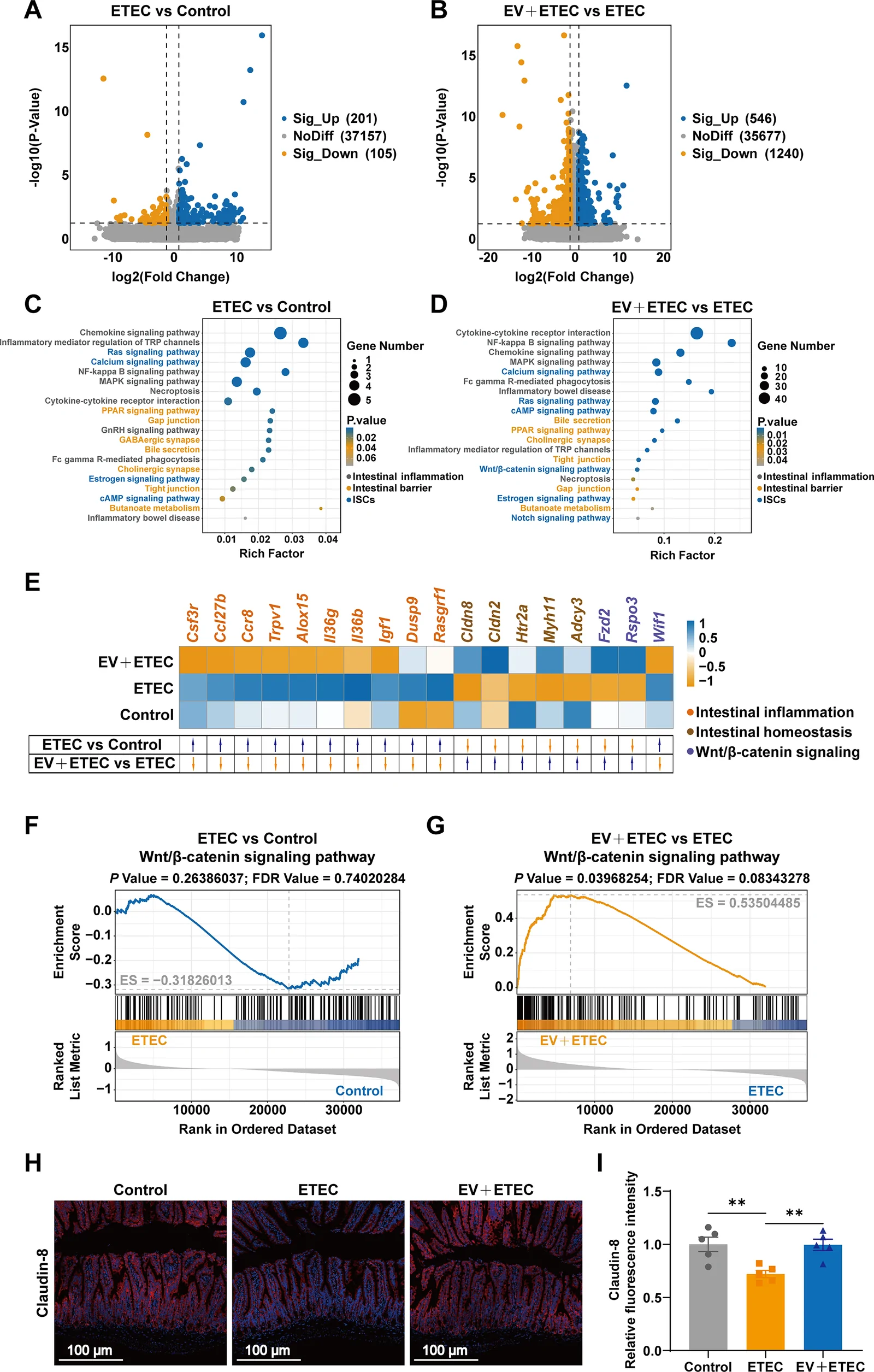
CbEVs activate Wnt/β-catenin signaling. (**A**) Volcano plot showing the DEGs between the ileal samples of the ETEC and control groups (fold change > 2, *P* < 0.05). (**B**) Volcano plot showing the DEGs between the ileal samples of the EV + ETEC and ETEC groups (fold change > 2, *P* < 0.05). (**C**) KEGG enrichment analysis of DEGs between the ileal samples of the ETEC *a*nd control groups (Gray font indicates signaling pathways related to intestinal inflammation, orange font indicates signaling pathways related to the intestinal barrier, and blue font indicates signaling pathways related to ISCs). (**D**) KEGG enrichment analysis of DEGs between the ileal samples of the EV + ETEC and ETEC groups (Gray font indicates signaling pathways related to intestinal inflammation, orange font indicates signaling pathways related to the intestinal barrier, and blue font indicates signaling pathways related to ISCs). (**E**) Heatmap of DEGs in the enriched pathways (Orange font indicates intestinal inflammation-related genes, brown font indicates intestinal homeostasis-related genes, and purple font indicates Wnt/β-catenin signaling-related genes). (**F**) GSEA showing the enrichment of DEGs in the Wnt/β-catenin signaling pathway between the ETEC and control groups. (**G**) GSEA showing the enrichment of DEGs in the Wnt/β-catenin signaling pathway between the EV + ETEC and ETEC groups. (**H**) Representative immunofluorescence images of Claudin-8 staining in ileal sections (red: Claudin-8, blue: DNA, scale bar = 100 µm). (**I**) Quantitative analysis of the relative fluorescence intensity of Claudin-8. All data are presented as the mean  ±  SEM, n = 5, *P ≤ 0.05, **P ≤ 0.01.

To further investigate the mechanism underlying the protective effect of CbEVs against ETEC K88-induced ileal injury, transcriptome analysis was performed on ileal tissues. DEG analysis revealed that ETEC K88 infection significantly upregulated 201 genes and significantly downregulated 105 genes (*P* < 0.05, Fig. 2A). In contrast, CbEV pretreatment significantly upregulated 546 genes and significantly downregulated 1,240 genes compared with the ETEC group (*P* < 0.05, Fig. 2B).

To explore the biological functions associated with these DEGs, KEGG pathway enrichment analysis was conducted. The results showed that these DEGs were significantly enriched in 20 signaling pathways (*P* < 0.05), mainly involving intestinal inflammation, intestinal barrier function, and ISC regulation (Fig. 2C and D).

The DEGs in these significantly enriched pathways mentioned above were further visualized using a heatmap (Fig. 2E). CbEVs inhibited the expression of classic intestinal inflammation-related genes, including *Csf3r*, *Ccl27b*, *Ccr8*, *Trpv1*, *Alox15*, *Il36g*, and *Il36b*. In addition, *Igf1*, *Dusp9*, and *Rasgrf1*, which have been reported to be involved in intestinal inflammatory injury (26), negative regulation of MAPK signaling (27), and Ras/MAPK signaling (28), respectively, were significantly suppressed by CbEV treatment (Fig. 2E). Moreover, CbEVs promoted the expression of intestinal tight junction marker *Cldn8*, as well as genes of intestinal homeostasis, including *Htr2a*, *Adcy3*, and *Myh11*, which are associated with serotonin-mediated intestinal epithelial regulation (29, 30), cAMP-associated epithelial signaling pathways (31), and intestinal smooth muscle function (32), respectively (Fig. 2E). Meanwhile, CbEVs significantly increased the expression of *Cldn2,* which may further enhance water efflux and then promote ETEC-K88 clearance (33). Notably, CbEVs increased the expression of Wnt/β-catenin-related genes *Fzd2* and *Rspo3*, while suppressing the expression of *Wif1*, a Wnt signaling inhibitor, suggesting a potential activation of Wnt/β-catenin-mediated ISC regulatory pathways (Fig. 2E).

GSEA was further performed to evaluate Wnt/β-catenin pathway-level alterations. The GSEA results showed that Wnt/β-catenin signaling was restrained in the ETEC group (*P* = 0.2639, Fig. 2F), but reactivated in the EV + ETEC group (*P* = 0.0397, Fig. 2G), indicating that CbEVs can restore Wnt/β-catenin signaling following ETEC K88 challenge.

We further selected key genes to validate the transcriptomic findings using RT-qPCR. Consistent with the RNA-seq results, CbEVs significantly reduced the expression of the pro-inflammatory cytokines *Il36g* (*P* = 0.009) and *Il36b* (*P* = 0.006) (Fig. S1A), in addition to the Wnt/β-catenin signaling inhibitor *Wif1* (*P* = 0.009) in ETEC K88-induced mouse ileal tissues (Fig. S1C). Conversely, CbEVs significantly increased the expression of the tight junction protein *Cldn8* (*P* = 0.011) (Fig. S1B) and the ISC marker gene *Lgr5* (*P* = 0.034) (Fig. S1C) in ETEC K88-induced mouse ileal tissues. In addition, CbEVs markedly upregulated the expression of Wnt/β-catenin signaling activators *Fzd2* (*P* = 0.004) and *Rspo3* (*P* = 0.005) (Fig. S1C). Consistent with the quantitative changes, immunofluorescence staining demonstrated that CbEVs significantly increased the relative fluorescence intensity of Claudin-8 (*P* = 0.003) compared with the ETEC group (Fig. 2H and I). These results indicate that CbEVs may alleviate ETEC K88-induced ileal injury in mice through activation of the Wnt/β-catenin signaling.

### CbEVs promote ISC proliferation and differentiation

**Fig 3:**
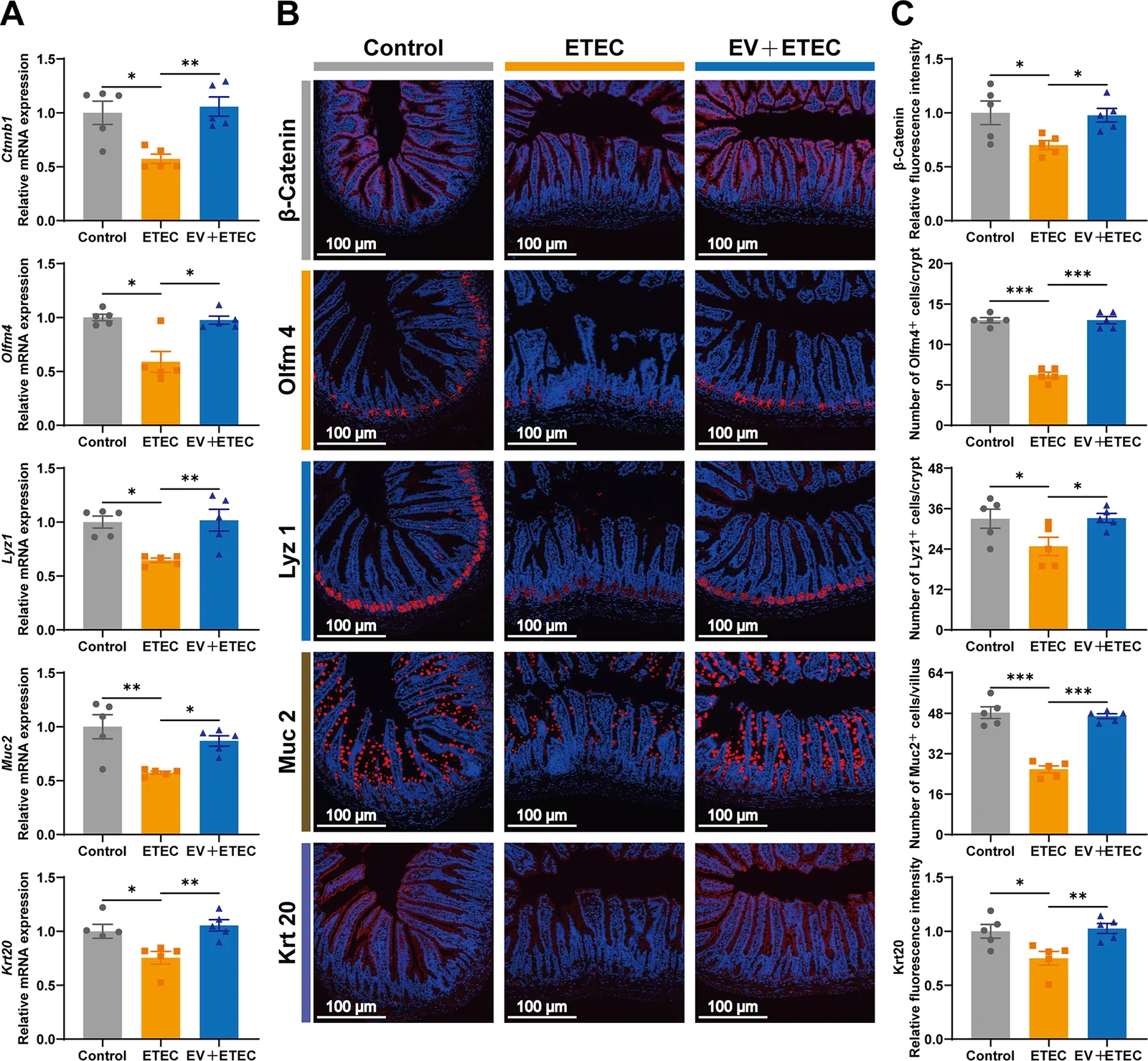
The effect of CbEVs on the proliferation and differentiation of ISCs. (**A**) Relative mRNA expression of *Ctnnb1*, *Olfm4*, *Lyz1*, *Muc2,* and *Krt20* in the ileal tissue was measured by RT-qPCR. (**B**) Representative immunofluorescence images of ileal sections stained with antibodies against β-catenin, Olfm4, Lyz1, Muc2, and Krt20 (red: β-catenin, Olfm4, Lyz1, Muc2, and Krt20, blue: DNA, scale bar = 100 µm). (**C**) Quantitative analysis of the relative fluorescence intensities of β-catenin and Krt20, and the numbers of Olfm4^＋^ and Lyz1^＋^ cells in ileal crypts as well as Muc2^＋^ cells in ileal villi. All data are presented as the mean  ±  SEM, n = 5, *P ≤ 0.05, **P ≤ 0.01, ***P ≤ 0.001.

Wnt/β-catenin signaling is a major regulator of ISC function (34). Thus, we subsequently explored whether CbEVs modulate ISC proliferation and differentiation in ETEC K88-infected mice. RT-qPCR showed that CbEVs significantly increased the relative mRNA expression levels of the Wnt/β-catenin signaling target gene *Ctnnb1* (*P* = 0.006), active ISC marker *Olfm4* (*P* = 0.047), Paneth cell marker *Lyz1* (*P* = 0.006), goblet cell marker *Muc2* (*P* = 0.020), and mature intestinal epithelial cell marker *Krt20* (*P* = 0.005) compared with the ETEC group (Fig. 3A). To further validate these findings, immunofluorescence staining was performed. Consistent with the RT-qPCR results, CbEVs significantly increased the relative fluorescence intensities of β-catenin (*P* = 0.016) and Krt20 (*P* = 0.006), as well as the numbers of Olfm4 (*P* < 0.001) and Lyz1 (*P* = 0.028) positive cells per crypt and Muc2 (*P* < 0.001) positive cells per villus in the ileum of ETEC K88*-*infected mice (Fig. 3B and C). Collectively, these results indicate that CbEVs may promote ISC proliferation and differentiation through activation of the Wnt/β-catenin signaling pathway.

### CbEVs modulate the composition of the ileal microbiota in ETEC K88-induced mice

**Fig 4:**
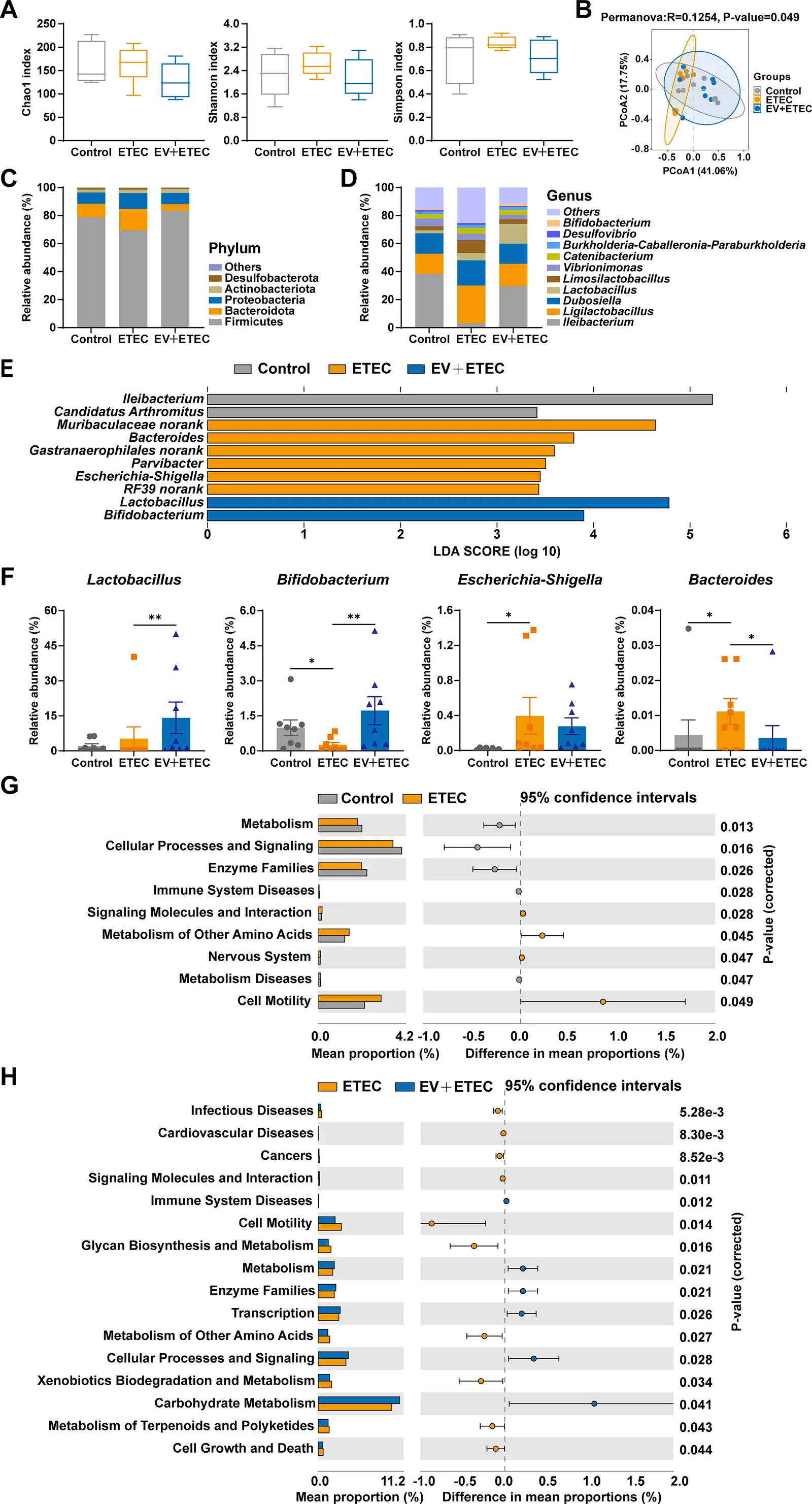
Effects of CbEVs on the ileal microbiota in ETEC K88-induced mice. (**A**) Chao1, Shannon, and Simpson indices in the α-diversity analysis. (**B**) β-diversity of each group was evaluated by PCoA. (**C**) Relative abundance of the top 5 ileal bacterial taxa at the phylum level. (**D**) Relative abundance of the top 10 ileal bacterial taxa at the genus level. (**E**) LEfSe analysis of the ileal microbiota at the genus level. (**F**) Relative abundance of differential genera in the ileum. (**G**) Functional prediction of the microbiota between the ETEC and control groups. (**H**) Functional prediction of the microbiota between the EV + ETEC and ETEC groups. All data are presented as the mean  ±  SEM, n = 8, *P ≤ 0.05, **P ≤ 0.01.

ETEC K88 infection is often accompanied by gut microbiota dysbiosis (35, 36). Therefore, 16S rRNA sequencing was performed to investigate whether CbEVs could reshape the ileal microbiota composition in ETEC K88-induced mice. α-diversity analysis showed no significant differences in the Chao1, Shannon, or Simpson indices among the groups (*P >* 0.05, Fig. 4A). PCoA revealed a clear separation of ileal microbial communities among the groups (*P* = 0.049, Fig. 4B). These findings indicate that CbEVs alter the composition of the ileal microbiota without substantially affecting overall diversity.

At the phylum level, Firmicutes, Bacteroidota, and Proteobacteria were the predominant bacterial phyla across all groups. Compared with the control group, ETEC K88 infection reduced the relative abundance of Firmicutes while increasing the relative abundance of Bacteroidota and Proteobacteria. Notably, CbEV treatment reversed these alterations (Fig. 4C). At the genus level, *Ileibacterium*, *Ligilactobacillus*, and *Dubosiella* were the predominant bacterial genera across all groups. Compared with the control group, ETEC K88 infection reduced the relative abundance of *Ileibacterium* while increasing the relative abundance of *Ligilactobacillus* and *Dubosiella*. These changes were also reversed by CbEV treatment (Fig. 4D).

LEfSe analysis was further performed to identify differential genera significantly enriched among the different treatment groups (Fig. 4E). CbEV treatment significantly increased the relative abundance of the probiotic genera *Lactobacillus* (*P* = 0.009) and *Bifidobacterium* (*P* = 0.006) in the ileum of ETEC K88-induced mice. Conversely, CbEV treatment reduced the relative abundance of the pathogenic genera *Escherichia-Shigella* (*P* = 0.524) and *Bacteroides* (*P* = 0.030) (Fig. 4F). These results indicate that CbEVs can alleviate the ileal microbial dysbiosis caused by ETEC K88 infection.

PICRUSt2 was used to predict the potential functional profiles of the ileal microbiota. Functional prediction revealed that metabolism, cellular processes and signaling, metabolism of other amino acids, immune system diseases, signaling molecules and interaction, enzyme families, and cell motility were common pathways among the three experimental groups. Compared with the control group, ETEC K88 infection significantly decreased the abundance of pathways related to enzyme families (*P* = 0.026), cellular processes and signaling (*P* = 0.016), and metabolism (*P* = 0.013) (Fig. 4G). Conversely, compared with the ETEC group, CbEV treatment significantly increased the abundance of pathways related to enzyme families (*P* = 0.021), cellular processes and signaling (*P* = 0.028), and metabolism (*P* = 0.021) (Fig. 4H). Furthermore, ETEC K88 infection significantly increased the abundance of cell motility-related pathways (*P* = 0.049) compared with the control group (Fig. 4G), whereas CbEV treatment significantly decreased the abundance of cell motility-related pathways (*P* = 0.014) compared with the ETEC group (Fig. 4H). Taken together, these results suggest that CbEVs may alleviate ETEC K88-induced intestinal dysfunction by modulating the functional potential of the ileal microbiota, thereby contributing to the maintenance of intestinal homeostasis.

### Gut microbiota and Wnt/β-catenin signaling are significantly correlated with ETEC K88*-*induced ileal injury

**Fig 5:**
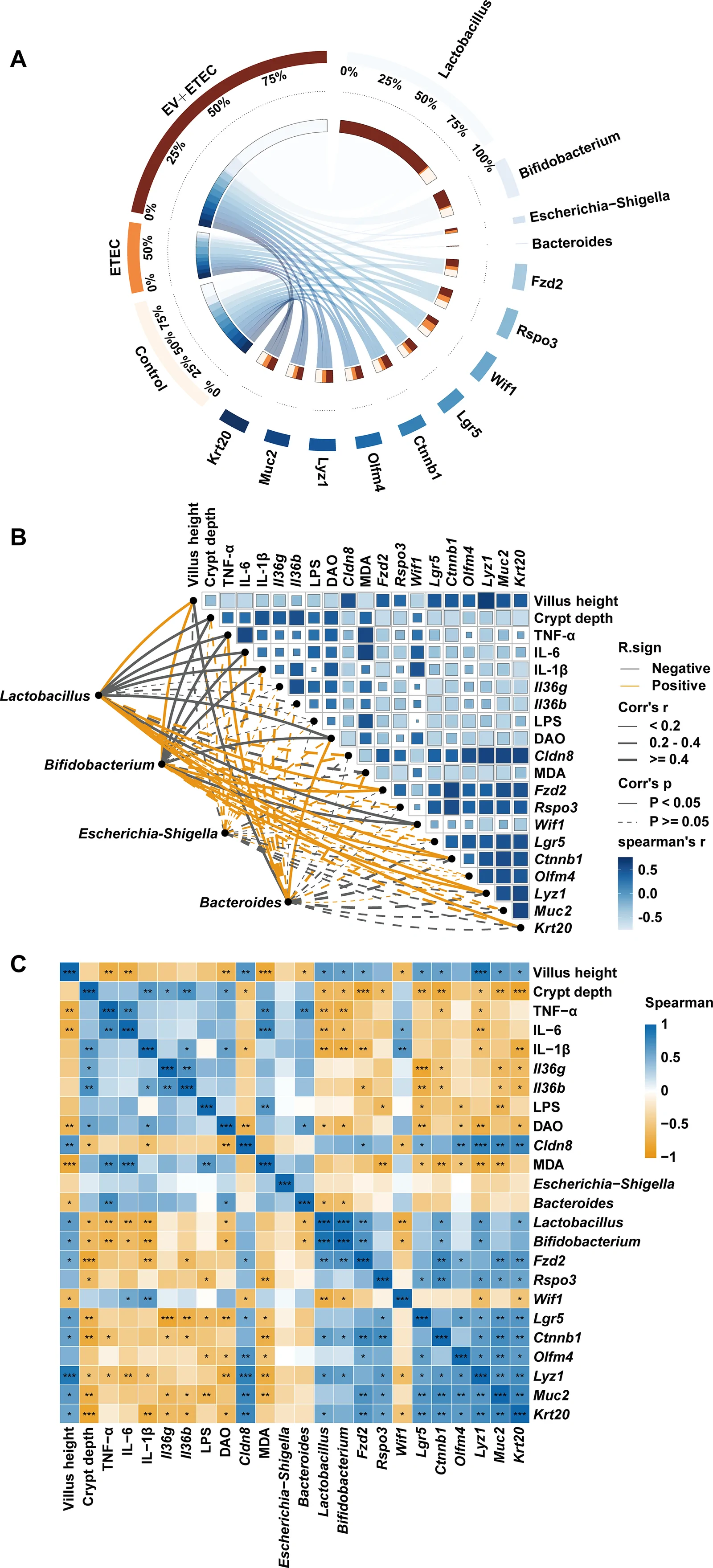
Correlation analysis of the gut microbiota, Wnt/β-catenin signaling, and ileal injury-related indicators. (**A**) Circos analysis showing the relationships between the gut microbiota and Wnt/β-catenin signaling in each group. (**B**) Correlation test revealing the associations among the gut microbiota, Wnt/β-catenin signaling, and ileal injury-related indicators. The color of the lines indicates positive or negative correlations, the thickness of the lines represents the absolute value of the correlation coefficient, and the line type indicates statistical significance. The gradient color bar represents the Spearman correlation coefficients for paired comparisons between Wnt/β-catenin signaling and ileal injury-related indicators. (**C**) Heatmap correlation analysis of the gut microbiota, Wnt/β-catenin signaling, and ileal injury-related indicators. All data are presented as the mean  ±  SEM, n = 5, *P ≤ 0.05, **P ≤ 0.01, ***P ≤ 0.001.

We further assessed the relationships among CbEV-mediated alleviation of ileal injury, alterations in gut microbiota composition, and Wnt/β-catenin signaling activity using correlation analysis. Circos analysis revealed that the distribution percentages of *Lactobacillus*, *Bifidobacterium*, *Escherichia-Shigella*, *Bacteroides*, *Fzd2*, *Rspo3*, *Wif1*, *Lgr5*, *Ctnnb1*, *Olfm4*, *Lyz1*, *Muc2,* and *Krt20* in the ETEC group were 1.37%, 6.16%, 53.20%, 50.00%, 20.37%, 27.48%, 39.00%, 21.88%, 21.79%, 22.94%, 24.25%, 23.50%, and 26.86%, respectively (Fig. 5A). In the EV + ETEC group, the corresponding distribution percentages were 87.03%, 62.45%, 44.17%, 22.41%, 41.29%, 39.03%, 27.04%, 39.14%, 40.20%, 38.06%, 38.20%, 35.55%, and 37.55%, respectively (Fig. 5A). Furthermore, correlation network heatmap analysis revealed that *Lactobacillus* and *Bifidobacterium* were positively correlated with villus height (*P* < 0.05) and *Cldn8.* However, these genera were negatively correlated with crypt depth (*P* < 0.05), IL-1β (*P* < 0.05), IL-6 (*P* < 0.05), TNF-α (*P* < 0.05), *Il36g*, *Il36b*, LPS, DAO (*P* < 0.05), and MDA (Fig. 5B). In contrast, *Escherichia-Shigella* and *Bacteroides* were negatively correlated with villus height and *Cldn8*, but positively correlated with crypt depth, IL-1β, IL-6, TNF-α, *Il36g*, *Il36b*, DAO, and MDA (Fig. 5B). Collectively, the protective effects of CbEVs against ileal injury are closely associated with the modulation of specific bacterial taxa, particularly *Lactobacillus*, *Bifidobacterium*, *Escherichia-Shigella*, and *Bacteroides*.

Heatmap correlation analysis revealed that *Fzd2*, *Rspo3*, *Lgr5*, *Ctnnb1*, *Olfm4*, *Lyz1*, *Muc2*, and *Krt20* were positively correlated with villus height and *Cldn8*, whereas they were negatively correlated with crypt depth, IL-1β, IL-6, TNF-α, *Il36g*, *Il36b*, LPS, DAO, and MDA (Fig. 5C). In contrast, *Wif1* was negatively correlated with villus height and *Cldn8*, but positively correlated with crypt depth, IL-1β, IL-6, TNF-α, *Il36g*, *Il36b*, DAO, and MDA (Fig. 5C). Taken together, these results indicate that CbEVs alleviate ETEC K88-induced ileal injury in mice by reshaping ileal microbiota composition and regulating ISC function via activation of the Wnt/β-catenin signaling pathway.

## DISCUSSION

Recent studies have shown that probiotic-derived EVs play important roles in alleviating intestinal inflammation (37). For example, EVs derived from *Lactobacillus plantarum* and *Lactobacillus rhamnosus* GG could suppress the production of pro-inflammatory cytokines, thereby mitigating colitis (38, 39). In this study, we demonstrated that CbEVs notably reduced the expression levels of IL-1β, IL-6, and TNF-α. Furthermore, EVs have been reported to facilitate the synthesis of tight junction proteins, thereby promoting epithelial repair and preventing pathogens and antigens from entering the body to trigger inflammation. For example, EVs derived from *E.coli* Nissle 1917 could notably ameliorate intestinal barrier injury and resist intestinal inflammation (40). Similarly, our results revealed that CbEVs notably reduced the expression levels of LPS and DAO, suggesting enhanced intestinal barrier function and decreased intestinal permeability. Moreover, as the end product of lipid peroxidation, MDA is widely recognized as a biomarker of oxidative stress and has been associated with the pathogenesis of ulcerative colitis (41, 42). In this study, CbEVs notably reduced the expression level of MDA. These results indicate that CbEVs alleviate ETEC K88-induced ileal injury in mice, as evidenced by reduced inflammation, improved intestinal barrier integrity, and attenuated oxidative stress.

Our study demonstrated that CbEVs activated Wnt/β-catenin signaling by upregulating the expression of the Wnt/β-catenin signaling activators *Fzd2* and *Rspo3*, while reducing the expression of the inhibitor *Wif1*. Consistent with our findings, EVs derived from *Lactobacillus paracasei* and *Lactobacillus reuteri* could promote osteogenic lineage commitment of mesenchymal stem cells and suppress osteoclast activity by activating Wnt/β-catenin signaling (43). Despite these findings, the specific bioactive components within CbEVs responsible for activating Wnt/β-catenin signaling remain largely unknown. Functioning as crucial vehicles for intercellular communication, EVs contain various bioactive substances, such as proteins, microRNAs, mRNAs, and lipids (44). Previous studies have shown that EVs could activate Wnt/β-catenin signaling by carrying Wnt ligands and microRNAs (45, 46). Future studies should focus on identifying the specific microRNAs or Wnt ligands within CbEVs responsible for activating Wnt/β-catenin signaling.

The dynamic balance between gut symbiotic bacteria and pathogenic bacteria is crucial for maintaining intestinal homeostasis (47). Previous studies have shown that EVs derived from probiotics could alleviate colitis by restoring gut microbiota homeostasis (18, 48). Consistent with these findings, our results revealed that CbEVs reduced the abundance of *Escherichia-Shigella*. Notably, CbEVs decreased predicted microbial functions related to cell motility, which are known to contribute to the colonization and virulence of pathogenic *Escherichia coli* (49). This finding may partially explain the reduced abundance of *Escherichia-Shigella* following CbEV treatment.

In addition to suppressing potentially harmful bacteria, CbEVs promoted the enrichment of *Lactobacillus* and *Bifidobacterium*, two well-recognized probiotic genera known to contribute to intestinal homeostasis. As key mediators of host-microbe interactions, EVs contain hydrolytic enzymes that facilitate the degradation of complex polysaccharides and proteins, thereby providing essential nutrients for microbial community growth (50). Moreover, EVs can regulate intestinal pH, redox status, and short-chain fatty acid production, which may create a favorable environment for probiotic colonization (51, 52). Therefore, CbEVs may facilitate the growth and colonization of beneficial bacteria, thereby contributing to the restoration of intestinal homeostasis.

Previous studies have shown that *Lactobacillus*- and *Bifidobacterium*-derived lactate can promote ISC self-renewal and differentiation by activating Wnt/β-catenin signaling, thereby maintaining intestinal epithelial regeneration and homeostasis (53, 54). Furthermore, *Lactobacillus* and *Bifidobacterium* could further enhance epithelial barrier integrity by modulating the gut microbiota and associated metabolites, thereby alleviating ulcerative colitis (55). In contrast, *Escherichia-Shigella* and *Bacteroides* were notably enriched in patients with Crohn’s disease (56). Therefore, CbEVs may mitigate ETEC K88-induced ileal injury in mice by reducing pathogenic bacteria and promoting probiotic enrichment.

However, this study also has several limitations. One limitation of this study is that heat-inactivated CbEVs and EV-depleted filtrates were not included as negative controls. Future studies incorporating these controls are needed to further distinguish EV-associated effects from effects potentially mediated by soluble extracellular factors and to determine whether heat-sensitive EV cargos contribute to the protective effects of CbEVs. Moreover, the key functional molecules responsible for these effects have not yet been identified. Future studies should identify the functional molecules in CbEVs using proteomic and metabolomic approaches and validate their roles through synthetic biology approaches. Furthermore, this study mainly revealed associations between gut microbiota alterations, Wnt/β-catenin signaling, and ileal injury. The causal relationship should be further confirmed through fecal microbiota transplantation experiments to determine whether microbiota remodeling is required for the protective effects of CbEVs.

### Conclusion

Overall, this study demonstrated that CbEVs effectively alleviated ETEC K88*-*induced ileal injury in mice. The protective effects of CbEVs were associated with gut microbiota remodeling and activation of the Wnt/β-catenin signaling pathway, thereby contributing to the maintenance of intestinal epithelial homeostasis. These findings provide novel insights into the therapeutic potential of CbEVs, and offer a theoretical basis for the application of EVs as effective strategies for the prevention and treatment of ETEC K88-induced intestinal injury.

## Data Availability

Transcriptomic data have been deposited in the NCBI Sequence Read Archive under accession number PRJNA1452193. The 16S rRNA gene sequencing data have been deposited in the NCBI Sequence Read Archive under accession number PRJNA1452084.

## SUPPLEMENTAL MATERIAL

Figure S1 aem.00424-26-s0001.tif Validation of key transcriptomic results in mouse ileal tissue by RT-qPCR.

Supplemental legend aem.00424-26-s0002.docx Descriptive legend for Fig. S1.

Graphical abstract aem.00424-26-s0003.tif CbEVs alleviate ETEC K88-induced intestinal injury.

Table S1 aem.00424-26-s0004.docx Primer sequences used for RT-qPCR in this study.

ASM does not own the copyrights to Supplemental Material that may be linked to, or accessed through, an article. The authors have granted ASM a non-exclusive, world-wide license to publish the Supplemental Material files. Please contact the corresponding author directly for reuse.
