# Supplementary material for: Clostridium butyricum-derived extracellular vesicles alleviate ETEC K88-induced intestinal injury by regulating Wnt/β-catenin signaling and gut microbiota
Source: Appl Environ Microbiol. 2026 Aug 28;92(9):e00424-26. doi: 10.1128/aem.00424-26 (PMC13599551; doi:10.1128/aem.00424-26)
Supplement: Supplementary file 1 [file aem.00424-26-s0002.docx]

**Figure S1** Validation of key transcriptomic results in mouse ileal tissue by RT-qPCR. (A) Relative mRNA expression of inflammation-related genes *Il36g* and *Il36b*. (B) Relative mRNA expression of the tight junction-related gene *Cldn8*. (C) Relative mRNA expression of Wnt/β-catenin signaling-related genes (*Fzd2*, *Rspo3*, *Wif1*) and ISC marker *Lgr5*. All data are presented as the mean ± SEM, n = 5, * *P* ≤ 0.05, ** *P* ≤ 0.01.
