# Supplementary material for: Clostridium butyricum-derived extracellular vesicles alleviate ETEC K88-induced intestinal injury by regulating Wnt/β-catenin signaling and gut microbiota
Source: Appl Environ Microbiol. 2026 Aug 28;92(9):e00424-26. doi: 10.1128/aem.00424-26 (PMC13599551; doi:10.1128/aem.00424-26)
Supplement: Supplementary file 2 [file aem.00424-26-s0004.docx]

**Table S1. The primer sequences used for RT-qPCR in this study**

| Target gene | Sequence (5’-3’) | | Genbank accession | Product size(bp) |
| --- | --- | --- | --- | --- |
| *β-Actin* | F:  R: | AGCCATGTACGTAGCCATCC  CTCTCAGCTGTGGTGGTGAA | NM_007393.5 | 228 |
| *Il36g* | F:  R: | CAGCAGGTGTGGATCTTTCG  ATAGTCACGCTGACTGGGGTT | NM_153511.3 | 80 |
| *Il36b* | F:  R: | TGATGGCTTTCCCTCCACAA  GGGAGATTGTGAGGATCCATGC | NM_027163.4 | 104 |
| *Cldn8* | F:  R: | AAGGTCTACGACTCCCTGCT  CATTCCGAGGATGGCTGTCA | NM_018778.3 | 114 |
| *Fzd2* | F:  R: | TGCTTCGTGGGCCTCAATAG  TGCTTCATGATGGTGCGGAT | NM_020510.2 | 137 |
| *Rspo3* | F:  R: | ACACCTTGGAAAGTGCCTTGA  CTGGCCTCACAGTGTACAATACT | NM_028351.3 | 93 |
| *Wif1* | F:  R: | ACCATCCTTAGGACCCCTCA  GGTATGCACAGGGCTTTCTCA | NM_011915.2 | 161 |
| *Lgr5* | F:  R: | CCTTCACAGCCTCAAAGTG  GCAGGGATTGAAGGCTTCT | NM_010195.2 | 95 |
| *Ctnnb1* | F:  R: | ATGGAGCCGGACAGAAAAGC  CTTGCCACTCAGGGAAGGA | NM_001165902.2 | 108 |
| *Olfm4* | F:  R: | CAGCCACTTTCCAATTTCACTG  GCTGGACATACTCCTTCACCTTA | NM_001351947.1 | 175 |
| *Lyz1* | F:  R: | AGGAATGGAATGGATGGCTA  CGGTCTCCACGGTTGTATT | NM_013590.4 | 110 |
| *Muc2* | F:  R: | ACGATGCCTACACCAAGGTC  TGATCTTCTGCATGTTCCCA | NM_023566.4 | 210 |
| *Krt20* | F:  R: | GTCCCACCTCAGCATGAAAGA  TCTGGCGTTCTGTGTCACTC | NM_023256.2 | 146 |
